# Ultrasound Imaging of Artificial Tongues During Compression and Shearing of Food Gels on a Biomimetic Testing Bench

**DOI:** 10.1111/jtxs.70030

**Published:** 2025-06-09

**Authors:** Miodrag Glumac, Jean‐Luc Gennisson, Vincent Mathieu

**Affiliations:** ^1^ Université Paris‐Saclay, INRAE, AgroParisTech, UMR SayFood Palaiseau France; ^2^ Laboratoire d'imagerie biomédicale multimodale (BioMaps) Université Paris‐Saclay, CEA Orsay France; ^3^ INRAE, Institut Agro, STLO Rennes France

**Keywords:** artificial tongue, food gels, food oral processing, ultrasound imaging

## Abstract

Characterizing the deformations undergone by the tongue during food oral processing could help to better understand how texture sensations are perceived. In this article, we propose to study the potential of ultrasound (US) imaging to monitor the deformations undergone by artificial tongues during compression and shear of agar food gels. Four polyvinyl alcohol cryogels were used as artificial tongues (two levels of roughness and two levels of stiffness), while three agar gels of different concentrations were considered as model foods. Throughout the experiments, US images were acquired from a transducer array positioned underneath the artificial tongue, while force signals were obtained from a multi‐axes sensor located above an artificial palate plate. Image analysis first consisted of tracing the contour of the dorsal surface of the artificial tongue. It was thus possible to observe how the deformations are distributed between the artificial tongues and the agar gels and to follow over time the heterogeneity of this distribution along the axis of the transducer array. Then, Particle Image Velocimetry (PIV) analysis was conducted to characterize the velocity fields related to deformations within the artificial tongue. In particular, the horizontal component of the velocity was studied during the shear movements and allowed one to distinguish static and dynamic friction phases, and to highlight the deformation gradients in the bulk of the artificial tongue. Such US method can provide a better understanding of the impact of the mechanical properties of food gels on the stimulation of mechanoreceptors responsible for translating mechanical stimuli into sensory perceptions.

## Graphical abstract

The mechanical breakdown of food gels during compression and shear on an artificial tongue‐palate system was observed with ultrasound imaging. Image processing methods of surface contouring and particle image velocimetry made it possible to monitor the deformations of the artificial tongue.

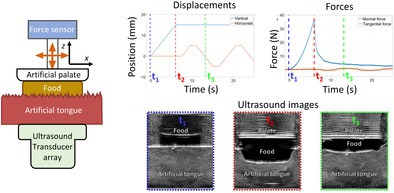

## 1 Introduction

Food oral processing (FOP) is the multidisciplinary study of how food is broken down and manipulated in the mouth during eating (Chen 2009). In recent decades, sensory perceptions related to the mechanical breakdown of food during FOP have been investigated thoroughly (Chen 2014; Chen and Engelen 2012). In particular, texture perceptions have been highlighted as one of the main drivers of food preferences and choices of consumers (Szczesniak 2002). The foods that make up our diets can be found in different physical states, and food gels have become excellent models for research dealing with structural breakdown during eating (Khalesi et al. 2020; Nishinari et al. 2019). By playing on the nature and concentration of the polymers that compose them, model food gels can be designed with a variety of physical properties (rheology, shape, size, structure) that may greatly affect texture perceptions, making them excellent objects of study for both in vitro and in vivo investigations. In the vast majority of cases, the oral breakdown of food gels mostly involves the tongue and the hard palate. The movements accomplished by the tongue (mixture of compressions and shear) result in complex fields of mechanical stresses and deformations, for which improved tools and methods of evaluation become necessary (Nishinari et al. 2019). The underlying challenge is to understand better the mechanical stimuli (both their intensity and their frequency) responsible for triggering tongue mechanoreceptors.

Food texture has been investigated on various instrumental setups, including texture analyzers, rheometers, as well as tribometers (Glumac 2024). A clear emerging trend consists of studying the mechanical behavior of foods on devices that allow taking into account the complexity of oral physiology, such as oral processing simulators (Andablo‐Reyes et al. 2020; Avila‐Sierra et al. 2024). In our group, a tongue‐palate biomimetic testing bench has been developed, capable of both compression and shear movements, all by accounting for some characteristics of the tongue (rigidity, roughness, lubrication) (Glumac 2024; Glumac et al. 2023; Srivastava, Bosc, et al. 2021). Such tools aim to contribute to understanding better the mechanisms of texture perception all by accounting for the variability of oral physiology. The objective is to be able to meet the specific needs of certain individuals with altered physiological functions (due to aging or to health issues). This is particularly the case for the growing part of the population affected by swallowing disorders (Adkins et al. 2020). Dysphagia may originate from various causes, such as a lack of lubricity due to insufficient salivary secretion (xerostomia) or even a loss of muscular strength (sarcopenia). In the field of food science, an important challenge consists of adapting to physiological constraints so as to be able to offer foods that are both safe and appreciated by people (Cichero et al. 2017).

To meet these challenges, the consideration of physiologically relevant environments is not sufficient. The development of original monitoring instruments is also critical to study the biomechanical interactions between the tongue, the palate, and the food. Biometric testing benches provide ideal platforms for the development and implementation of methods for characterizing and monitoring these interactions. In particular, characterizing the deformations undergone by artificial tongues during experiments conducted on this type of device constitutes an important objective. Indeed, such a measurement would allow us to investigate the mechanical stimuli to which the mechanoreceptors responsible for sensory perceptions of texture are subjected. It would thus be possible to analyze the respective contributions of the properties of artificial tongues and foods on these stimuli, both during compressional and shearing actions. To date, there is no method available to achieve this objective, and most of the work described in the literature is devoted to the study of the fracture behavior of food. Some studies proposed video‐capturing devices to characterize the breakage of food gels during uniaxial compression on artificial soft tongues (Kohyama 2020). Ultrasound (US) methods with non‐destructive and non‐invasive mono‐element transducers have also been proposed in several studies to investigate the breakage of food gels during uniaxial compressions (Gao et al. 2016; Mantelet, Srivastava, et al. 2020; Srivastava, Mantelet, et al. 2021).

An in vivo study has shown the interest and potential, but also the high complexity of US B‐mode images of the tongue to follow and quantify food oral processing of semi‐solid food of varied viscosity (de Wijk et al. 2006). US imaging of the tongue also has been utilized in in vivo studies with research topics other than food oral processing, such as for the study of sleep apnea (Weng et al. 2017), swallowing (Kwong et al. 2022), or also human speech (Al‐hammuri et al. 2022). For this last area, the importance of being able to extract the movements of the tongue has been underlined, and image processing methods have been developed to monitor the contour of the dorsal surface of the tongue on US images (Akgul et al. 1999). The Active Contour Model, commonly known as “snake”, is a technique used in image processing and computer vision to detect and delineate objects within an image. The Active Contour Model was introduced by Kass et al. (Kass et al. 1988), relying on the basic principle of a deformable curve that evolves within an image data set to conform to the edges of objects of interest. The curve is influenced by internal and external forces that guide it toward the object's boundaries. Such a method has been applied to isolate and map tongue contours from US images (Laporte and Ménard 2018; Li et al. 2005). This method offers an interesting alternative to more complex machine learning approaches which have also been implemented (Al‐hammuri et al. 2022; Jaumard‐Hakoun et al. 2016; Li et al. 2022; Zhu et al. 2019), but which require rigorous and meticulous work for the manual annotation of large data sets to be used for learning.

Additionally, the evaluation of the fields of deformation in the bulk of the artificial tongues can provide valuable information on the spatial distribution of the mechanical stresses applied during the experiments. Particle Image Velocimetry (PIV) can be a promising method to reach this objective, consisting of defining a region of interest in images and dividing it into small interrogation windows used for cross‐correlation calculations between consecutive frames. This determines the average particle displacement within each window.

The main objective of the present article is to assess the feasibility of acquiring, processing, and analyzing ultrasound images to derive information on the fields of deformation of artificial tongues during compression or shearing of food gels. To do so, the article describes the different stages of the development and the validation of an ultrasound method implemented on our biomimetic testing bench. The first criterion for the success of the study was to manage to transform the acquired ultrasound images into semi‐quantitative forms in order to be able to describe and compare the kinetics of the deformations of the artificial tongues in different experiments. To do so, the two image analysis methods introduced here before were implemented on US imaging data sets: the active contour model method focused on tracking the upper surface of the artificial tongue, while particle image velocimetry was applied to characterize fields of velocity in the bulk of the artificial tongue. The second criterion for the success of the study was to manage to implement the two image processing methods in image sets from contrasting experimental conditions. Varying properties in artificial tongues (roughness, stiffness) and in food gels (agar concentration) led to contrasting mechanical behaviors on the biomimetic testing bench (in terms of elastic deformation, fracture, shear, and friction). This provided an adequate framework for evaluating the robustness of image processing.

Thanks to the feasibility of such methodological development, it will be possible, in the future, to focus more precisely on scientific questions linked to the mechanical interactions between tongue and food. This work may thus broaden the range of tools available to assess the respective contributions of tongue and food properties to the mechanisms underlying texture perception.

## 2 Materials and Methods

### 2.1 Food Gels

Three different gels were considered as model foods in the study, differing in their concentration in agar (Ag), the main polymer used in their formulation. The preparation followed a previously described protocol (Srivastava, Mantelet, et al. 2021). In short, sucrose (Daddy, CristalCo SAS, Paris, France) was added and dissolved in ultrapure water at 10% g/l (w/w) and underwent stirring at room temperature (20°C) for 30 min. Then, edible agar (SAS Nature and Plants, Magescq, France) was added to the water and sucrose solution in concentrations of 0.45%, 0.60%, and 1.00% g/l (w/w) and heated at 95°C for 45 min under continuous magnetic stirring, with it being covered with aluminum foil. After this, it was poured into silicone molds (ELASTOMOULE, De Buyer, Le Val‐d'Ajol, France) and kept in the refrigerator at ~5°C for a duration of 18 h. Two types of silicone molds were used: cylindrical molds were used to design samples suitable for mechanical characterizations, while cubical ones were considered to prepare samples dedicated to experiments on the biomimetic test bench. Cylindrical samples had a diameter of 25 mm and a height of 20 mm, while cubical ones had dimensions equal to 25 × 25 × 20 mm^3^ (width, depth, and height). Before the experiments, food gels were left at room temperature for 15 min and taken out from their silicone molds. After this, cylindrical gels went through a compression test using a texture analyzer (TA.XT plus, Stable Micro Systems, Surrey, United Kingdom). The samples were subjected to a deformation of 20% (relative to their initial height) at the speed of 10 mm/s. Stress vs. strain slope was characterized for the estimation of the Young's modulus (between 2.5% and 5%, over which linear elasticity could be observed). Five replicates were performed for each of the three types of gels. The composition of the three different gels and the obtained values of Young's modulus are summarized in Table 1. The gels are referred to according to their concentration in agar: Ag_0.45_, Ag_0.60_, and Ag_1.00_.

**TABLE 1:** Composition and Young's modulus (average ± standard deviation) of food gels.

Label	Composition (% w/w)			Young's modulus (kPa)
	Water	Agar	Sugar	
Ag_0.45_	89.55	0.45	10.00	6.45 ± 0.38
Ag_0.60_	89.40	0.60	10.00	7.97 ± 0.28
Ag_1.00_	89.00	1.00	10.00	18.35 ± 0.83

### 2.2 Artificial Tongues

A total of four artificial tongues were manufactured and used in the study, varying both in terms of roughness and rigidity. The roughness of the artificial tongues was varied to account for the topography of the tongue surface, which is primarily induced by the filiform papillae that cover the dorsal surface. In previous work, roughness properties were found to strongly affect the mechanical interactions between the artificial tongue and food, both during compression (Srivastava, Stieger, et al. 2021) and shear experiments (Glumac et al. 2023). Furthermore, this roughness greatly affects the transmission of ultrasound waves at the tongue‐food interface, due to air bubbles trapped in between (Mantelet, Restagno, et al. 2020). It was therefore all the more important to take this factor into account in the present study.

The artificial tongues were Polyvinyl Alcohol (PVA) cryogels, prepared according to previously established protocols (Srivastava, Mantelet, et al. 2021; Srivastava, Stieger, et al. 2021). PVA powder (MW 89,000–98,000, 99% hydrolyzed, Sigma Aldrich, Saint‐Louis, USA) was dissolved in ultrapure water at 10% (w/w) under magnetic stirring for 2 h at 80°C. After this, the temperature of the solution was cooled down to room temperature (20°C). In the present study, the main change made to the protocol consisted of adding 1% (w/w) cellulose particles with a size distribution of 63–126 μm (Cellets 90, IPC GMBH & Co., Dresden, Germany) during the cooling step under continuous magnetic stirring. The scattering properties of these particles make it possible to increase the contrast of ultrasound images and to better observe the fields of deformation in the bulk of the artificial tongues. The obtained solution was poured into rectangular molds of 80 × 45 × 25 mm^3^ (width, depth, and height), the lower surface of which was covered with sandpaper to roughen the corresponding surface of the artificial tongue. Two references of sandpaper (Leman, Saint‐Clair‐de‐la‐Tour, France) were used (P_100_ and P_36_), leading to artificial tongues surfaces hereafter referred to as “smooth” or “rough”. Profilometry characterizations performed in previous works (Glumac et al. 2023) made it possible to establish that the grit size of the sandpaper (550 μm for P_36_, 160 μm for P_100_) turns out to correspond approximately to the average width of the asperities (RSm parameter) of the obtained artificial tongues. In terms of roughness average height (Ra parameter), artificial tongues prepared with the same two types of sandpaper (P_100_ and P_36_) resulted in average asperity heights of 25 and 140 μm, respectively (Glumac et al. 2023). These values were found to match with data collected in vivo on human tongues, ranging from 20 to 120 μm (Uemori et al. 2012).

The molds were kept at −20°C for 16 h and thawed at 20°C for 8 h. The rigidity of the artificial tongues was varied by adapting the number of cycles of freezing and thawing undergone by the samples: two cycles for the samples referred to as “compliant”, four cycles for those referred to as “hard”. Finally, the artificial tongues were unmolded and immersed in water using plastic containers, hermetically sealed, and kept at room temperature. The rigidity of the artificial tongues was characterized following the same protocol as for the food gels: 20% strain rate at the speed of 10 mm/s. Young's modulus was determined from the stress/strain slope between 2.5% and 5.0%. The average and standard deviations of the Young modulus after five replicates were 25.57 ± 0.76 kPa for compliant artificial tongues and 47.01 ± 0.80 kPa for hard artificial tongues. The four obtained artificial tongues are labeled as follows: T_H/S_, T_H/R_, T_C/S_, T_C/R_ (H: hard, C: compliant; S: smooth; R: rough, respectively).

### 2.3 Tongue‐Palate Biomimetic Testing Bench

#### 2.3.1 Description of the Device

**FIGURE 1:**
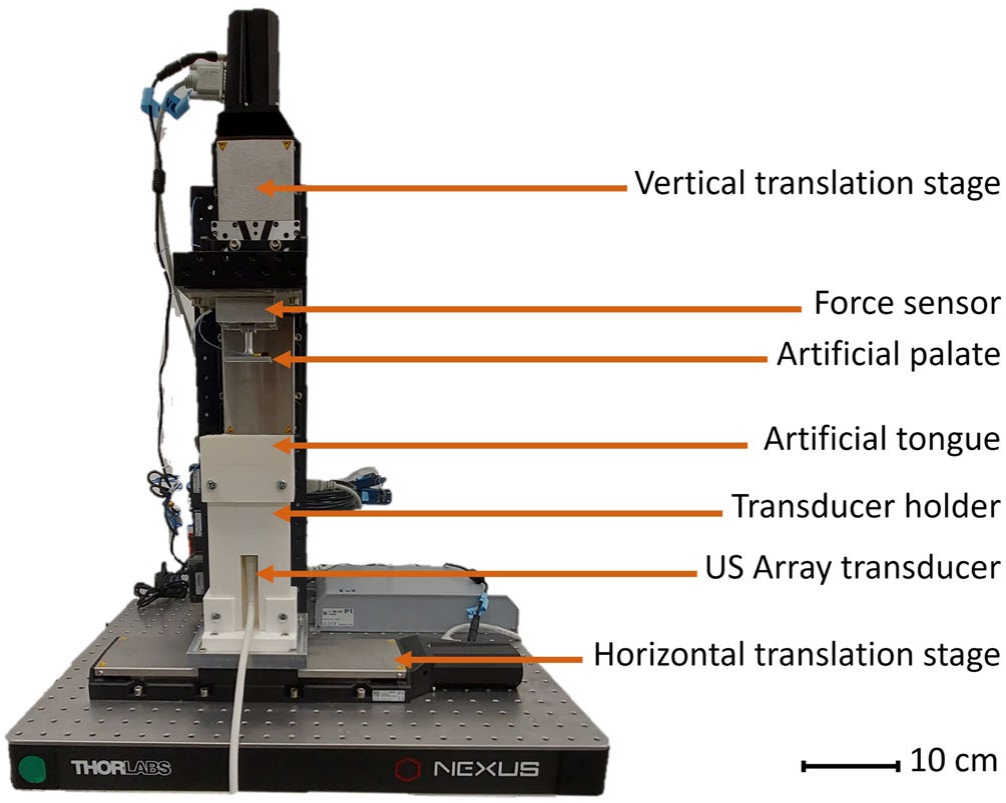
Picture of the tongue‐palate biomimetic testing bench, with the description of the main components.

The artificial tongues described in the previous section were implemented on a tongue‐palate biomimetic testing bench, an illustration of which is provided in Figure 1. The system was described in detail in our previous article and consists of two orthogonal translation stages (Glumac et al. 2023). A rigid and smooth plate (25 × 45 mm^2^), mimicking the hard palate, is fixed on the vertical stage. Similarly to what was done in our previous papers, a multi‐axes force sensor (K3D60a, ME Systeme, Hennigsdorf, Germany) was positioned between the artificial palate and the vertical translation stage to measure the evolution of the normal and tangential forces as a function of time (Srivastava, Bosc, et al. 2021). The sensor has a measuring range of ±50 N, with an accuracy of 1%, and a bridge module with a 25 kHz sampling rate (NI‐9237, N.I., Texas, USA) was used for data acquisition. The artificial tongue is for its part inserted into a holder fixed on the horizontal stage.

#### 2.3.2 Experimental Procedure

The protocol and measurements performed during each test are described hereafter. The experiments were conducted at room temperature (20°C), as the current version of the biomimetic setup does not yet enable temperature control. Food gels were taken out from refrigerators 10 min before launching a test. Then, the artificial tongue was taken from the water container in which it is stored, and the excess of water was absorbed with paper (KIMTECH Science, Kimberly‐Clark Europe Limited, Surrey, UK). After this, the cube‐shaped food gel was unmolded and positioned in the middle of the artificial tongue. The aluminum artificial palate was lowered until it just touched the top part of the food gel. After having reached this position, an automatic sequence of imposed displacements was launched. The advantage of imposing the same sequence of movements (magnitude and speed) in each experiment is that it facilitates the comparability of results with synchronized data.

Few experimental devices allow for the simultaneous consideration of compression and shear movements between an artificial tongue and palate. Our biomimetic testing bench was designed with this in mind, offering the ability to custom‐impose movement sequences combining these two types of movements. In this first study, where we consider compression and shear in the same sequence, the choice was made to separate them for the sake of simplicity. The decision was to begin with uniaxial compression, which is an effective way to fracture the gel, and then continue with shear cycles similar to those performed in our previous studies (Glumac et al. 2023). By doing so, different critical steps of tongue‐palate oral strategy associated with this kind of food products were roughly reproduced.

**FIGURE 2:**
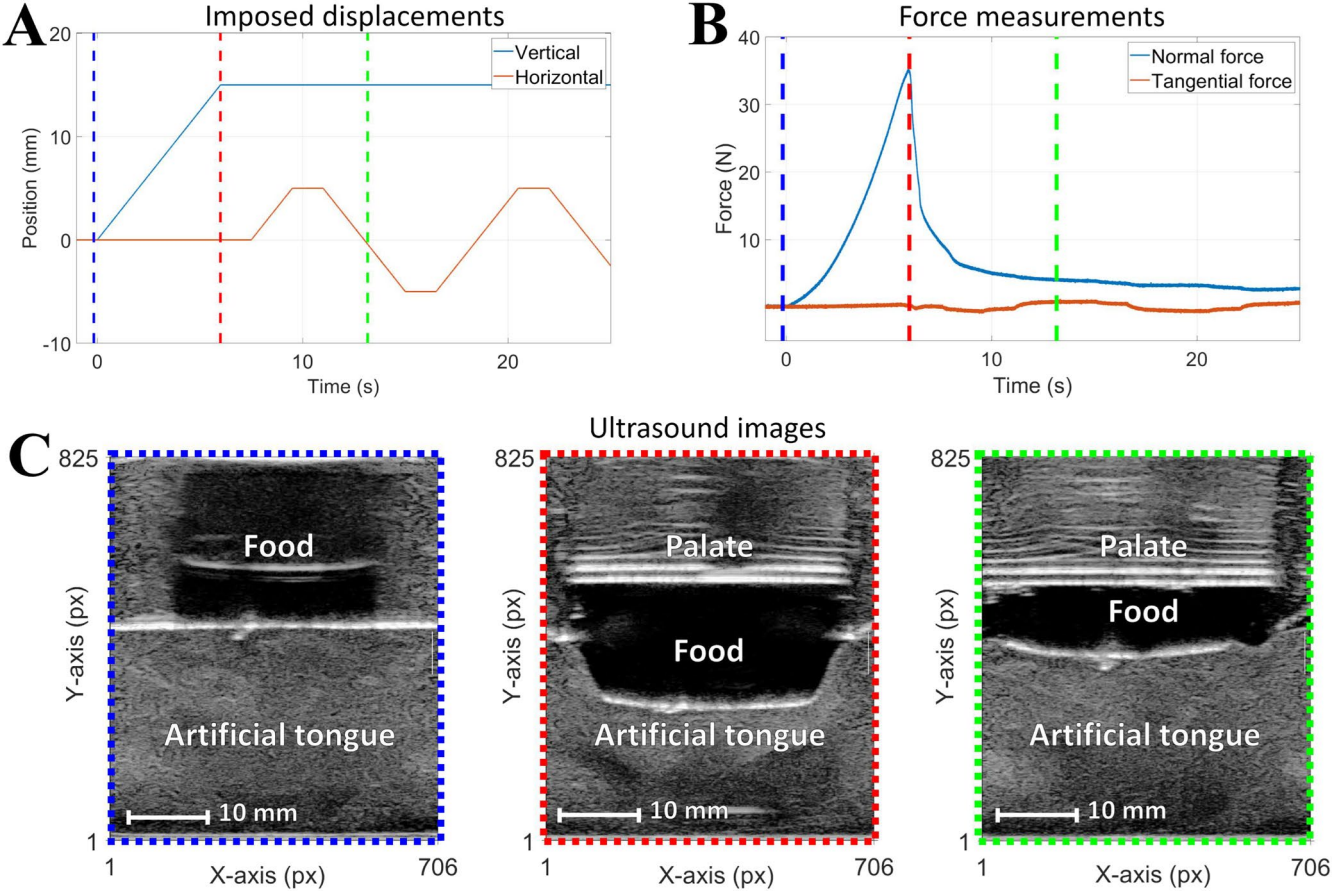
(A) Displacements of the vertical (blue solid line) and horizontal (orange solid line) translation stages as a function of time during a test protocol. (B) Corresponding variations of the normal (blue solid line) and tangential (orange solid line) forces were measured with the multi‐axes force sensor (Artificial tongue: T_C/S_; Food gel: Ag_1.00_). (C) US images were acquired at different times during an experiment. The colors of the lines surrounding each US image match with those of the vertical dotted lines in (A) and (B): Before the start of compression (blue), at the end of compression (red), during a shearing motion (green).

Figure 2A shows the movements of the vertical and horizontal stages operated as a function of time during the sequence imposed for all experiments. The displacement of the vertical stage (represented with a blue line) during the compression step was 15 mm (equivalent to a strain rate of 75%), reached at a constant speed of 2.5 mm/s. After this, the horizontal stage (motions represented by the orange line in Figure 2A) operates four successive shearing motions at the same constant velocity of 2.5 mm/s. The first motion has an amplitude of 5 mm, against 10 mm for the three others, this in order to maintain movements centered in relation to the initial position of the food sample. Each movement of one of the translation stages is followed by a rest period of 1.5 s. The displacement speeds of the stages were significantly reduced compared to what was done in the compression tests to characterize the Young's modulus (it was 10 mm/s). This may have led to an increase in the contribution of viscoelastic characteristics to the force signals (due to relaxation times). In return, this allowed us to observe more finely the details in the evolution of the ultrasound images, which were acquired at a frequency of 60 Hz.

### 2.4 Ultrasound Imaging

#### 2.4.1 Device and Acquisition Settings

The main evolution of the device lies in the design of a new adaptation part between the horizontal stage and the tongue holder. This part was 3‐D printed so that it fits the shape of a US transducer array, securing its position just underneath the artificial tongue. Ultrasound gel (Healthlife, Barclay Swann ltd, Long Sutton, UK) was used to improve the acoustic coupling between the transducer array and the artificial tongue. This product has been spread on the surface of the transducer array before inserting the artificial tongue in the holder. This step prevented the formation of air bubbles at the interface, which otherwise has the effect of significantly disrupting the propagation of ultrasound.

The US images were captured using a medical‐grade ultrafast US imaging device (Aixplorer, SuperSonic Imagine, Aix‐en‐Provence, France) equipped with a linear multielement transducer array (SL10‐2 SuperLinear, 6 MHz central frequency, 192 elements, SuperSonic Imagine, Aix‐en‐Provence, France). The transducer array was mounted on the oral tribometer with the help of a custom‐designed 3D‐printed part attached to the oral tribometer. An orifice made centrally at the bottom of the tongue holder allows the transducer array to be brought into contact with the artificial tongue (the coupling is ensured by US gel). “General” settings preset was used. The investigation depth was set to 25 mm. Focal depth cursor was positioned at the upper surface of the artificial tongue. US videos were captured in a resolution of 1440 by 1080 with a 60 Hz framerate.

The *X*‐axis (width) of ultrasound images reflects the position along the 192 elements that make up the transducer array. The *Y*‐axis (depth) corresponds for its part to the time elapsed between the emission of ultrasound waves and their return to the transducer array after having been reflected at an interface. Based on a velocity of 1540 m/s^−1^, which is the propagation speed in a 10% PVA gel (Fromageau et al. 2007), the device provides a conversion indication of 183 pixels for 10 mm. The 2‐D image is constructed so that the *X* (width) and *Y* (depth) axes have equivalent spatial resolutions. The conversion ratio (identical in all ultrasound images in the article) is provided for information purposes only (as a good order of magnitude), and all images remain drawn in pixels.

#### 2.4.2 Image Processing

The sets of images composing the videos from US acquisitions went through two main analysis processes. The first one had for aim to capture the evolution of the profile of the contour of the dorsal surface of the artificial tongue as a function of time. For the second one, the objective was to visualize and characterize the fields of deformation in the bulk of the artificial tongues. Figure 2C shows three examples of US images acquired, each one corresponding to a specific time during a test. On all of them, the analysis was focused on the gray region in the bottom part, which corresponds to the artificial tongue. The lower end of the images, which delimits this region at the bottom, corresponds to the surface of the US transducer array in contact with the artificial tongue. The white line which demarcates the upper part of this region, more or less curved depending on the images, delimits the interface between the dorsal surface of the tongue and the environments in contact with it. Most of the image analysis described hereafter deals with tracking either this dorsal surface or the fields of deformation below this surface. Of course, the rest of the image is also of great interest, in particular to characterize the mechanical behavior of the food located between the tongue and the palate during compression and shearing movements. In the present article, this mechanical behavior is investigated in an indirect way, by quantifying the impact of food mechanical properties on the response observed on the artificial tongue.

##### 2.4.2.1 Tongue Contour Extraction

**FIGURE 3:**
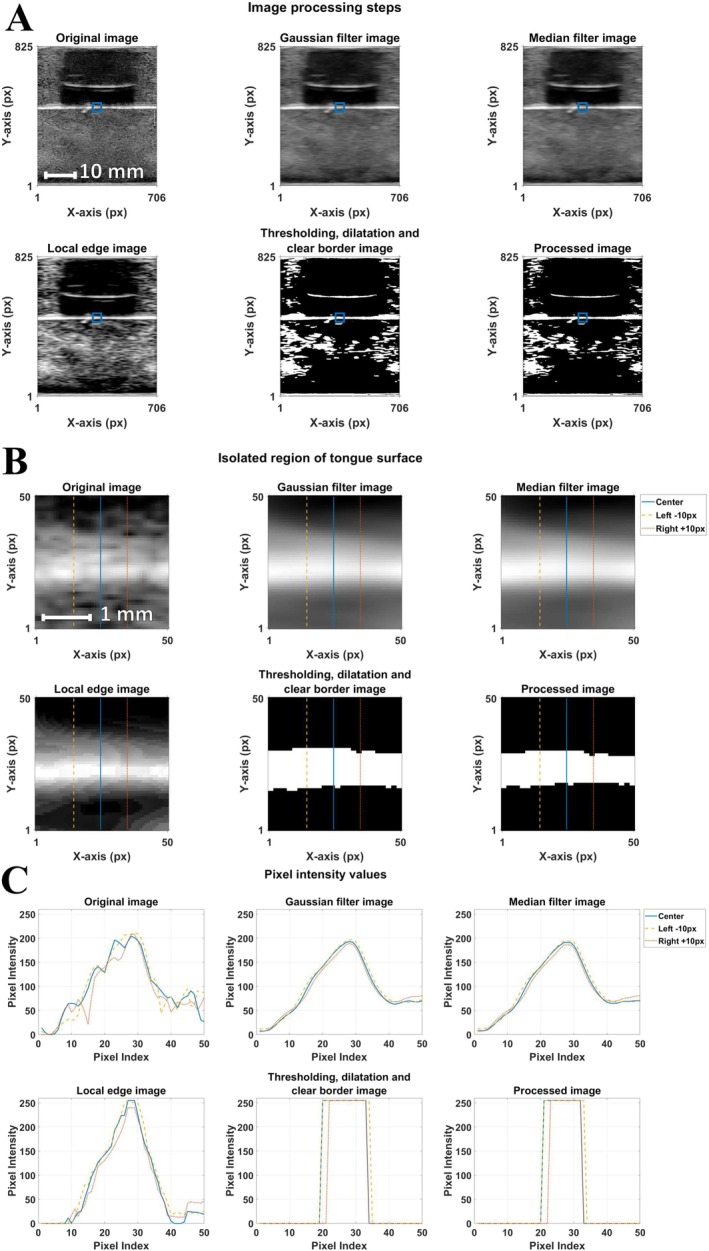
(A) Different steps of image analysis illustrated with an experiment combining an artificial tongue T_C/S_ with a food gel Ag_1.00_. (B) Isolated region of artificial tongue surface (size of 50 px × 50 px, corresponding to the blue squares in A) during different steps of image analysis. Three vertical lines indicate the locations of the calculations of pixel intensity values represented in (C) for the different steps of image analysis that were applied to the images.

Each frame composing the US videos acquired during the experiments underwent several steps of image processing to make it more suitable for tongue contour tracking. These steps were chosen based on thorough testing and adjusting to our dataset. Implementing these filters is common for medical US imaging in order to achieve desired processing that can be case‐specific. In our dataset, the main aim was to improve the contrast at the top border of the artificial tongue, to make it easier to detect. Finally, all the steps are described: gaussian filter with a kernel size of 2 px per 10 px; median filter with a kernel size of 5 px per 5 px; edge thresholding by 0.6, followed by thresholding (150–225); dilatation step with three dilatation objects (line: size 1, 90°; line: size 1, 45, degrees: disk, size 1); clear border step with the erosion step done to polish the pixels from the tongue surface (diamond: size 1) (Figure 3A). To better visualize the impact of image processing, a region of 50 × 50 px^2^ (illustrated with a blue square in Figure 3A) was isolated from the middle point of the image (in the region of the artificial tongue surface) and represented in Figure 3B. In this image, three vertical lines were selected to extract the pixel intensity values: from the middle point of the image, 10 px to the left from the middle point, and 10 pixels to the right from the middle point. The results of the pixel intensity values are shown in Figure 3C. Here, we can see values ranging from 0 to 225 on the *y*‐axis and the 50 pixels region on the *x*‐axis. Progressively implementing filters is noticeable and presented in the changes in pixel intensity values. From the first original image subplot, irregularities in the pixel intensity values are noticeable for all three regions, which are shown to be smoothed out in the second figure with the Gaussian filter image. After this, the medial filter enabled us to form and later isolate a small plateau with index values of around 27 to 29 pixels, which was then found to have a maximum level of 255 intensity in the next figure of the local edge image. After the thresholding, dilatation, and clear border image, we have binary values with an artificial tongue surface image isolated from 20 to 33 pixel index values. The final image has this region stabilized to 21–32 pixel index values, making it 11 pixels in width. This is a graphical representation of how the irregularities in the first image (Figure 3C, original image) were reduced to a clear and binary region found in Figure 3C processed image. This enabled the snake algorithm to properly recognize this tongue surface border for the whole duration of the sequence of motions and provide precise data on the surface of the artificial tongue behavior.

After all these steps, a MATLAB (The MathWorks, Natick, Massachusetts, USA) based open source platform called GetContours was used for evaluating the contours of the dorsal surface of the artificial tongue on each of the processed images. This platform, developed by Laporte and Ménard (2018), was designed specifically to meet the need for simple interactive tools that can be used to trace the tongue, in particular for research on speech (https://github.com/mktiede/GetContours). The method is based on click‐and‐drag positioning of reference points on the interface of interest, followed by the control of a fit by comparison with the analyzed US image. The fitting model that was used was a variation of the SNAKE algorithm (M. Li et al. 2005) called the “gtc_snake” algorithm in GetContours software. The algorithm parameters were the default ones: ‐colormap indicating the color scheme of the images: gray;‐sigma indicating the values of the Gaussian smoothing applied to the image: 5 and 2;‐delta indicating the step size of the resolution in which the algorithm operates: 2;‐band penalty that indicates how flexible the contour is to bending (higher values make the contour stiffer, lower values more flexible): 2;‐alpha that indicates a weight to the internal energy of the snake algorithm that controls the overall smoothness (high‐value likely to make contour smooth and not overfit small noise): 0.80;‐lambda that indicates a weight to the external energy of the snake algorithm and enables the contour's ability to be sensitive to attaching edges with higher values: 0.95 (https://github.com/mktiede/GetContours).

At the end of this first sequence of image analysis, a set of data is obtained, consisting of tables of the 2‐D coordinates of the dorsal surface of the artificial tongue, for each experiment. Each column in the table corresponds to the horizontal position, from one end to the other of the US transducer array axis (scaled from 1 to 100). Each row corresponds to a specific frame from the start to the end of an experiment, with, for each coordinate, the corresponding value of the time‐of‐flight obtained from the tongue contouring algorithm.

##### 2.4.2.2 Particle Image Velocimetry

**FIGURE 4:**
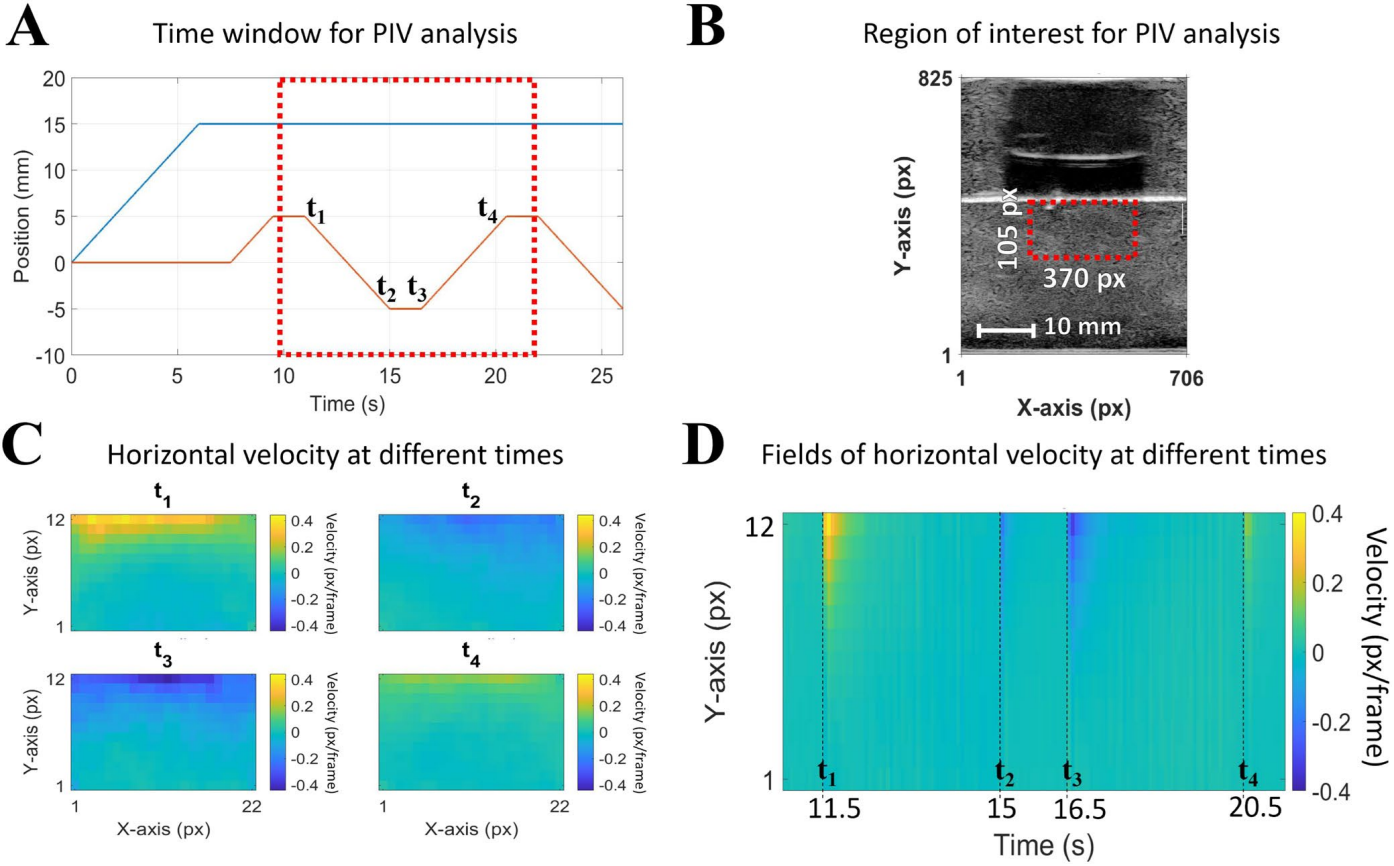
(A) Illustration of the time window (red dotted line) and of the specific time points (from *t*_1_ to *t*_4_) during which the Particle Image Velocimetry analyses were carried out to assess the fields of horizontal velocity during shear motions. (B) For an experiment conducted on artificial tongue T_C/S_ combined with food gel Ag_1.00_, an ultrasound image with the representation of the region of interest (ROI), demarcated by red rectangles. (C) Maps of horizontal velocities obtained by PIV at the four time points represented over the entire ROI. (D) Variations over time of the horizontal velocities obtained by PIV, represented over the form of average values along the *x*‐axis.

Particle image velocimetry was performed to visualize and quantify the fields of velocity associated with the deformations in the bulk of the artificial tongues. In particular, the aim was to study horizontal deformations (in response to shear motions), which were not observable with the first image analysis method described above. The analysis was thus performed from 10 to 22 s (as shown on a red background in Figure 4A), which corresponds to a time period during which only shear motions occur. The digital tool used for this purpose was PIVlab (https://github.com/Shrediquette/PIVlab), an open source software for Particle Image Velocimetry (PIV) running with MATLAB (The MathWorks, Natick, Massachusetts, USA) (Thielicke and Sonntag 2021). The US videos used for PIV analysis underwent image processing available in PIVlab, called contrast‐limited adaptive histogram equalization. Then, a rectangular region of interest (ROI) was defined, with identical dimensions (370 pixels in width; 105 pixels in height) for all the images from all the experiments (Figure 4B). The positioning of this ROI was adjusted individually for each experiment, such that its upper limit coincided with the dorsal surface of the artificial tongue. In this way, the vertical coordinates of the ROI are comparable from image to image and from experiment to experiment, corresponding from top to bottom the distance to the dorsal surface of the artificial tongue. The FFT window deformation algorithm was used. The interrogation area was set to 64 pixels for pass #1 and 32 pixels for pass #2, those settings having been defined after a series of pre‐tests to ensure that the size of the interrogation area respects the criteria of displacement amplitude, all by optimizing vector resolution. For each analysis, the output corresponds to sets of grids covering the ROIs. The image resolution of the grid depends on the size and overlap of the interrogation areas used in PIV analysis. In Figure 4 (C&D), the grid of the velocity field has dimensions of 22 pixels by 12 pixels. For each coordinate point of the grids, PIV analysis makes it possible to obtain the evolution of the vertical or horizontal component of velocity vectors. Figure 4A shows four time points at which PIV analysis was focused on: from *t*_1_ to *t*_4_, each time point corresponds to the first frame after having either initiated (*t*_1_ and *t*_3_) or ended (*t*_2_ and *t*_4_) a shearing motion. Figure 4C shows four heatmaps of horizontal velocities in the ROI, obtained from PIV analysis at times *t*_1_, *t*_2_, *t*_3_, and *t*_4_. Figure 4D provides another type of visualization of the results, still under the form of a heatmap of the horizontal velocity, but where the *X*‐axis corresponds to the time elapsed during the experiment and the *Y*‐axis remains the vertical position in the ROI. The velocity plotted in color corresponds to the average velocity value obtained over the entire width of the ROI.

## 3 Results and Discussion

This section is composed of two parts. In the first one, the temporal variations of the normal force measured in the different combinations of artificial tongues and food gels are discussed in light of the variations of US time‐of‐flight of the center of the dorsal surface of the tongue. The analysis is then extended to the whole width of the tongue contour through 2D plots. The second section focuses on PIV measurements, with a particular interest in the horizontal component of velocity.

### 3.1 Tongue Deformation Rates

**FIGURE 5:**
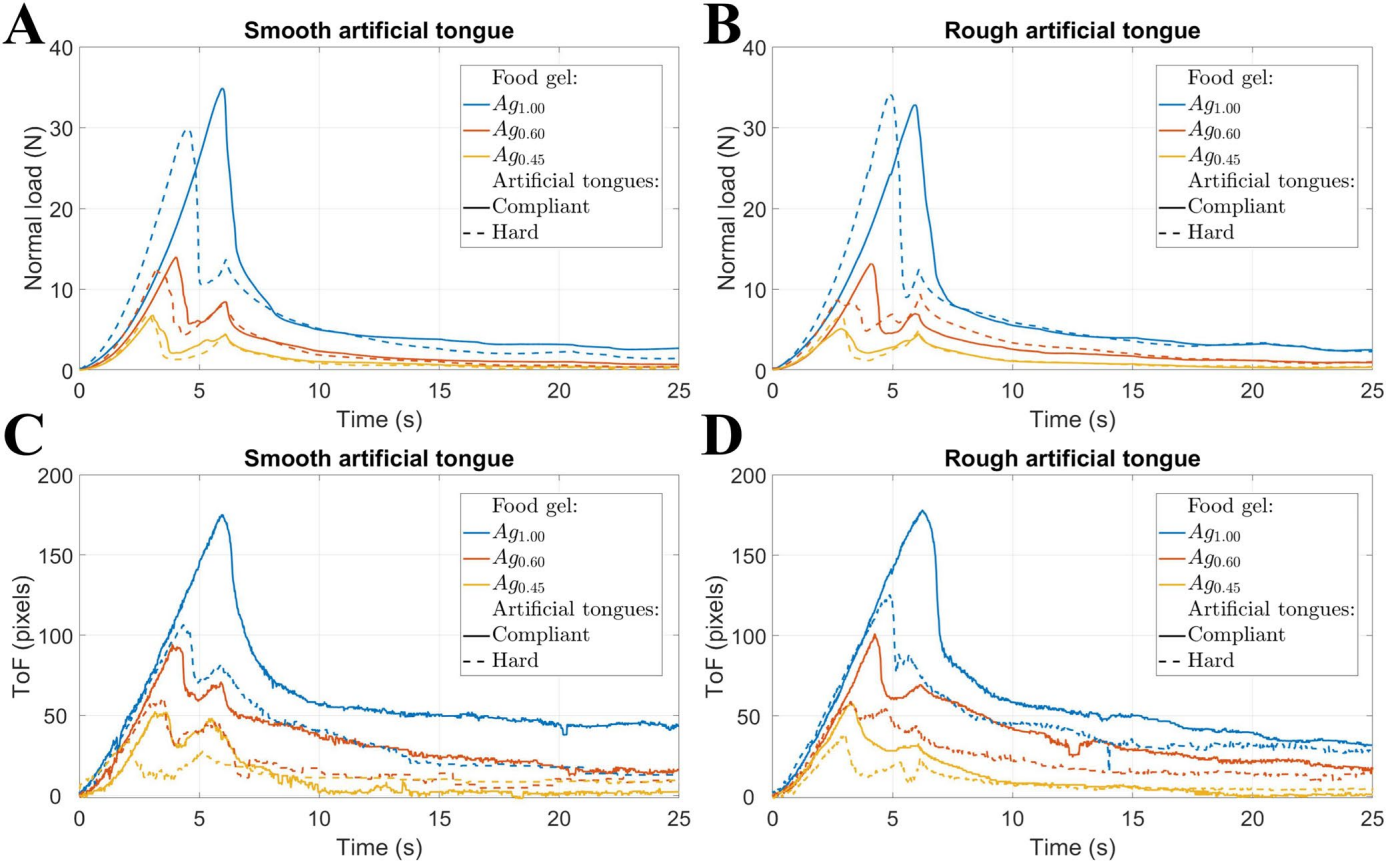
Evolution of the normal force measured as a function of time for the different combinations of artificial tongues and food gels: Experiments on smooth artificial tongues in (A), and rough ones in (B). Corresponding evolutions of the US time‐of‐flight measured at the level of the middle of the US probe: For smooth artificial tongues in (C) and for rough ones in (D). Each color is associated with a type of food gel: Blue for Ag_1.00_, orange for Ag_0.60_, and yellow for Ag_0.45_. The style of the line corresponds to the rigidity of the artificial tongue: Solid line for “compliant” artificial tongues, dashed line for “hard” ones.

In order to see how the mechanical behavior of the food gels influenced the deformation of the artificial tongue, force measurements were compared with the results of tongue surface tracking obtained from US imaging acquisitions. Figure 5A,B show the evolution of the normal force, measured as a function of time for the different combinations of artificial tongues and food gels: for all the experiments conducted on smooth artificial tongues (A), and on rough ones (B). Each color is associated with a type of food gel: blue for Ag_1.00_, orange for Ag_0.60_, and yellow for Ag_0.45_. The style of the line corresponds to the rigidity of the artificial tongue: solid line for compliant artificial tongues, dashed line for hard ones. In all of these experiments, the compression step of the protocol had a vertical displacement amplitude of 15 mm, reached with a constant velocity of 2.5 mm.s^−1^. Regardless of the type of artificial tongue they were paired with, the two softest gels (Ag_0.45_ and Ag_0.60_) fractured before the end of the compression stage (which is at *t* = 6 s). Similar behaviors were reported for the stiffer gel Ag_1.00_ when combined with a “compliant” tongue. However, when paired with a “hard” tongue (both for “smooth” and “rough” ones), the force peak coincided with the end of the compressional motion. The fracture thus resulted both from the stresses accumulated during compression and from the tearing of the gel under the effect of shearing initiation. Although the experiments presented here only include one test for each experimental configuration (three food gels combined with four artificial tongues), the observed behaviors follow the expected logic of the mechanical case of two springs in series. For the same level of total imposed deformation (15 mm amplitude), the more rigid the food gel, the greater the equivalent stiffness of the tongue‐food system and the greater the force measured in response. For the same food gel, the differences obtained between “compliant” and “hard” tongues were less observable in terms of the amplitude of forces reached than in the level of overall displacement to be imposed to observe gel fracture (which is less important for “hard” tongues). No clear trends could be observed concerning the impact of the roughness of the artificial tongues.

The different curves of the evolution of the normal force could be related to the US data resulting from tongue contour tracking. For each US image, a set of coordinates forming the contour of the dorsal surface of the tongue was obtained. The horizontal axis is graduated from 1 to 100 and covers the total width of the US images. The vertical axis corresponds to the extracted time‐of‐flight (ToF) of the echo formed by the reflection of US waves at the level of the dorsal surface of the tongue. This time is directly proportional to the distance between the surface of the US transducer array and the dorsal surface of the artificial tongue.

A first visualization of the results consisted of focusing on the evolution of the US time‐of‐flight measured at the graduation *x* = 50, which corresponds to the middle of the transducer array. Figure 5C,D show the different profiles of variations of the absolute values of US ToF as a function of time obtained on “smooth” and “rough” artificial tongues, respectively. The results show a qualitative agreement between the variations observed on the normal force and on the US ToF. This is particularly illustrated when we closely observe the synchronization of the peaks linked to gel fractures. The US traces therefore reflect the elastic behavior of the artificial tongues, in response to the forces they are exposed to. Contrastingly with what was observed on the curves of normal force, when we compare the amplitude of ToF variations during the fracture of identical gels on artificial tongues of different rigidity, we systematically note lower tongue deformations for “hard” than for “compliant” ones. The rigidity of the “compliant” artificial tongue being closer to that of the three food gels than the “hard” tongue, the deformation was distributed in a more balanced manner between the artificial tongue and the food in the case of “compliant” tongue. The results show the complementarity between force and deformation measurements obtained from the two modalities.

**FIGURE 6:**
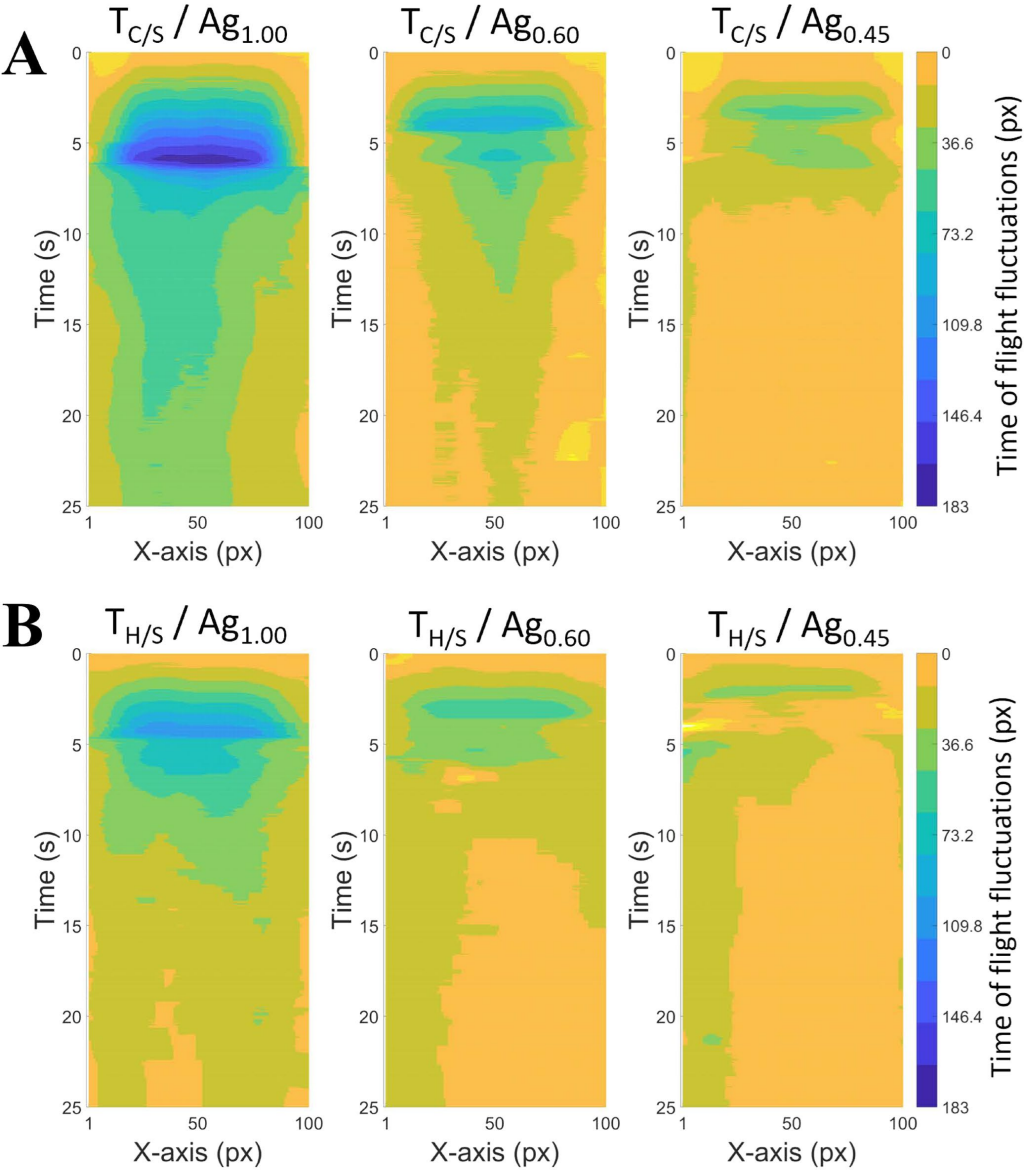
2‐D representations of the evolution of the time‐of‐flight of the echo of the US waves reflected on the top surface of the artificial tongues. Graphs correspond to one out of the three food gels, with the top row (A) being paired with a “compliant” and “smooth” artificial tongue while the bottom row (B) being paired with a “hard” and “smooth” artificial tongue. The horizontal axis represents the lateral position along the width of the US probe. The vertical axis, from 0 to 25.5 s, represents the time elapsed during an experiment. The intensity of time‐of‐flight fluctuations is color coded.

In addition to the previous analysis of tongue echo in the central axis of the US transducer array (as in Figure 5C,D), tongue contour evaluations were taken a step further and broadened to the entire width of the multielement US probe. The graphs displayed in Figure 6 provide 2‐D representations of the temporal evolution of the time‐of‐flight of the echo of the US waves reflected on the top surface of the artificial tongues. The horizontal axis, graduated from 1 to 100, represents the position across the width of the surface of the transducer array. The vertical axis, from 0 to 26 s, represents the time elapsed during an experiment. The colors describe the intensity of time‐of‐flight fluctuations, associated with the amplitude of the displacements of the artificial tongue surface: starting from yellow (no deformation) and gradually increasing to blue (maximum deformation). Figure 6 focuses on three experiments performed with each of the three food gels, all of them being paired with a “smooth” and “compliant” artificial tongue.

During the compression phase (between 0 and 6 s), a more pronounced intensity of deformations was confirmed in the case of the stiffer gel (Ag_1.00_). For the two softer samples (Ag_0.60_ and Ag_0.45_), the two‐stage fractures already reported were also observable. Prior to each fracture, the region on which the deformations are distributed systematically tended to broaden, reflecting the lateral spreading behaviors of the gels during compression. During the cycles of shearing motions following the compression step (from 7.5 to 26 s), various behaviors were reported between the three food gels in terms of decay in the deformation of the tongue. For the most rigid food gel Ag_1.00_, the intensity of the deformations and the width over which they are distributed decreased over time throughout the cycles of shearing motions. These decreases were even more marked in the case of the Ag_0.60_ food gel. On the other hand, for the softest food gel Ag_0.45_, the level of residual deformation was almost zero and no longer changed after the first shearing motion. Regardless of the type of gel, low levels of lateral shifts were reported in the area on which tongue deformations were concentrated. The 2‐D images resulting from these contour analyses are thus complementary to force measurements, offering a more complete understanding of the phenomena involved.

During the compression step, the fracture of agar gels may induce several factors of variability in the mechanical response of the system. In particular, the heterogeneity of the size and shape of resulting gel particles is likely to vary significantly from one experiment to another (even in identical conditions). Such variability may affect the spatial distribution of the deformations of the artificial tongue during the end of the compression phase, as well as during the shear movements that follow. In the absence of replications, the present study does not allow for the study of the variability of these factors. However, it is important to develop methodological solutions that can provide relevance for doing so, particularly all along shear movements during which the presence of gel fragments may affect tongue‐palate friction.

### 3.2 Tongue Velocity Fields

In order to characterize strain fields in the artificial tongues during shearing motions, particle image velocimetry was applied, and the displacement rates (expressed in pixels per frame) were followed over time. PIV analyses were carried out on rectangular regions of interest (ROIs) of 370 pixels in width and 105 pixels in height. These ROIs were meshed according to the size of the interrogation areas. The results of PIV analyses result in a grid of interrogated areas covering the entire ROI. In each interrogated area, PIV analysis results in the values of the horizontal and vertical components of the speeds (expressed in pixels/frame) obtained from the cross‐correlations of two consecutive frames in the sets of US images. Hereafter, the objective was to focus on horizontal velocities in order to investigate the potential of PIV to monitor deformations occurring in the bulk of the artificial tongue during shearing motions.

In the present experimental protocol, the horizontal movements occurred between 7.5 and 26 s. Most of the food gels have already been crushed during the compression, and the latter were crushed during the first shear. The resulting particles of the gels are distributed between the artificial tongue and palate.

**FIGURE 7:**
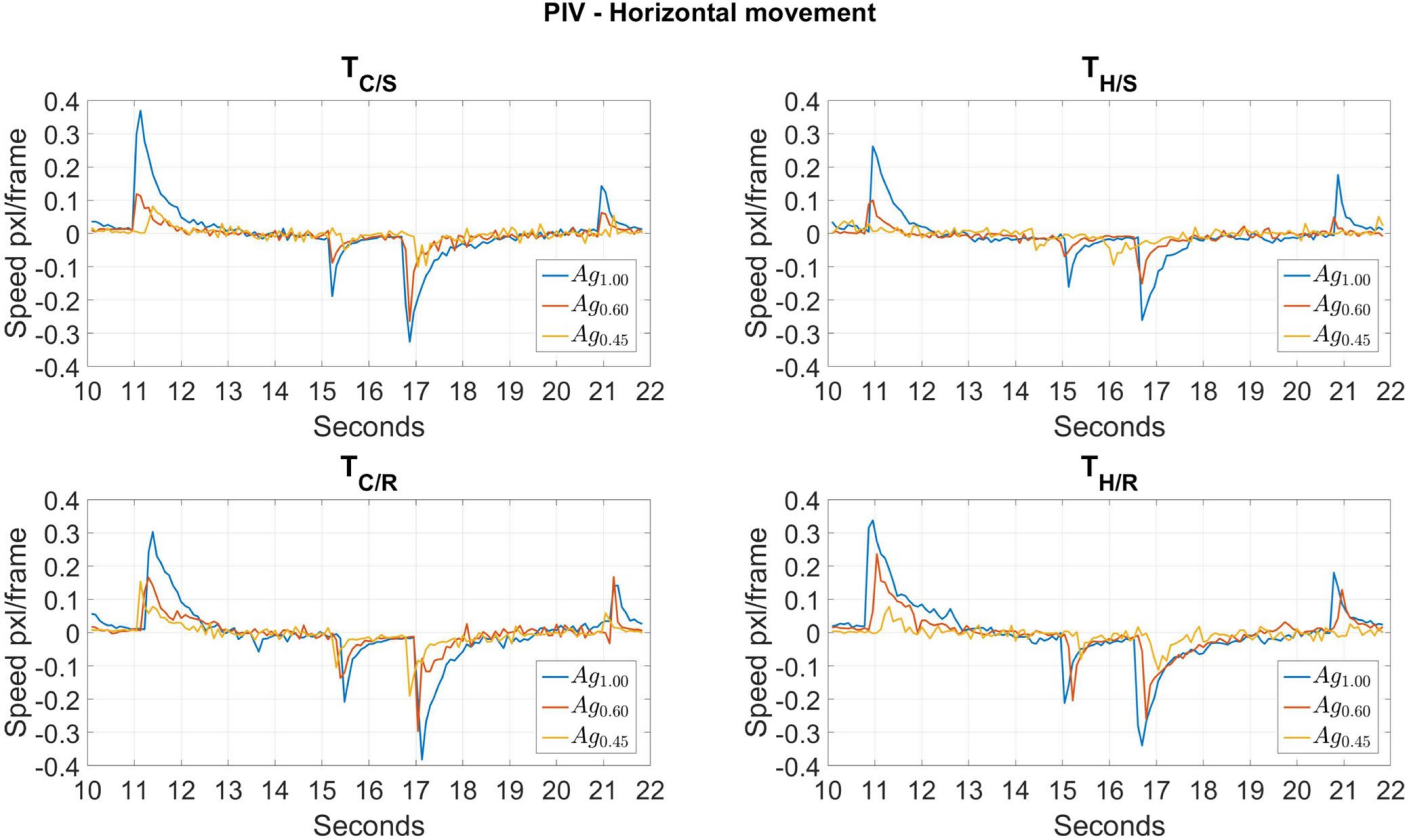
Temporal evolutions of the horizontal component velocities obtained from PIV analysis during shearing motions between 10 and 22 s. Represented values correspond to an interrogation area located laterally in the middle of the US probe, and in vertical coordinate as close as possible to the top surface of the artificial tongue.

Figure 7 shows the temporal evolutions of the horizontal component of velocities obtained from PIV analysis during shearing motions between 10 and 22 s. This interval was chosen because it contains a complete back‐and‐forth shearing motion, with symmetry with respect to the central axis of the artificial palate. Represented values correspond to an interrogation area located laterally in the middle of the US transducer array, and in the vertical coordinate as close as possible to the top surface of the artificial tongue. This velocity is the consequence of the mechanical stresses exerted on the surface of the artificial tongue during shearing against the artificial palate, in the presence of fragments of food gels. Positive and negative values of velocities indicate the direction of the motions. During the time window shown in Figure 7, two main events occur: a first shear in one direction (between 11 s and 15 s), followed by a second in the opposite direction (between 16.5 s and 20.5 s) to return to the starting point. It should be noted that, concomitantly with the beginning and end of the shear movements, velocity peaks can be observed. At *t* = 11 and 16.5 s, the measured velocities are the consequence of static friction, during which there is no relative displacement between the surfaces of the artificial tongue and palate. The velocity peaks thus correspond to the elastic deformations undergone by the artificial tongue. The sudden decrease observed just after crossing the velocity peak corresponds to the beginning of the dynamic friction phase, with sliding between the artificial tongue and palate that induces a decrease in velocity. At the moment when shear movements stop (at *t* = 15 and 20.5 s), new velocity peaks can be observed, of lesser intensity and with reversed polarity. These peaks reflect relative sliding between the artificial tongue and palate, during which the artificial tongue can release part of the energy stored during the elastic deformations it underwent during the shear. Generally, one can note that the peaks at the beginning of a movement tend to have a larger amplitude and duration than the peaks at the end of the movement. This suggests that there are residual deformations of the artificial tongue, before going to the next shearing movement.

Although the experiments performed under the different conditions (three food gels combined with four artificial tongues) were not repeated, it should be noted that with all of the four artificial tongues, the stiffest gel (Ag_1.00_) systematically led to the largest amplitudes of velocity peaks, followed by the longest decay phases. These observations thus seem to reflect more significant adhesion phenomena in the case of this gel, which we have also seen to lead to the highest levels of normal force. In contrast, the softest gel Ag_0.45_ is the one that displays the lowest intensity of velocity variation as a function of time. This observation would be consistent with low levels of friction between the tongue and the palate. There is no qualitative observation of the results that suggests a clear impact of the rigidity or roughness of the artificial tongues. Further experiments, with repetitions, will need to be conducted to allow reliable conclusions about these factors.

## 4 Conclusions

In summary, the preliminary experiments presented in this study show the encouraging potential of US imaging to monitor the deformations of artificial tongues during compression and shear movements in the presence of food gels. The image analysis methods implemented for tracking the dorsal surface of artificial tongues are promising. In particular, they allow access to the spatial distributions of tongue surface displacements, which are likely to vary depending on the capacity of food gels to spread at the interface, particularly during compression phases. PIV analyses allow monitoring deformations. If the analysis presented in this study focuses on velocities in coordinates as close as possible to the upper surface of the tongue, there will also be many possibilities in the long term to study velocity gradients along the vertical axis (toward the surface of the US transducer array). Such an US method can provide a better understanding of the impact of the mechanical properties of food gels on the stimulation of mechanoreceptors responsible for translating mechanical stimuli into sensory perceptions. Despite the absence of repetitions (linked to the preliminary nature of the study), clear trends are observed regarding the impact of the mechanical properties of gels. In future studies, a more complete experimental design is necessary. Quantitative indicators will need to be developed in order to translate some of the image analysis results presented here (deformation profiles, velocity mapping) into forms more suited to fully quantitative comparisons between the different experimental conditions. The work thus opens the way to numerous applications for understanding the biomechanical phenomena related to the perception of food texture. In particular, tongue movements are undoubtedly much more complex than the sequences of motions considered here. In future works, the logic of gradually increasing the complexity of the experimental conditions will be followed, for example, by considering mixed movements simultaneously combining compression and shearing and trying to get as close as possible to real oral strategies, which adapt to the characteristics of the food.

## Author Contributions

**Miodrag Glumac:** conceptualization, methodology, validation, formal analysis, investigation, resources, data curation, writing – original draft, writing – review and editing, visualization. **Jean‐Luc Gennisson:** conceptualization, methodology, writing – review and editing. **Vincent Mathieu:** conceptualization, methodology, software, validation, formal analysis, investigation, resources, data curation, writing – original draft, writing – review and editing, visualization, supervision, project administration, funding acquisition.

## Conflicts of Interest

Jean‐Luc Gennisson is a scientific consultant for the company Supersonic Imagine, which built up the ultrasound imaging device used in this study.

## Data Availability

The data that support the findings of this study are available from the corresponding author upon reasonable request.
